# The risk of sodium overcorrections in severe hyponatremia and the utility of desmopressin: a large retrospective study

**DOI:** 10.1093/ckj/sfae386

**Published:** 2024-11-29

**Authors:** Florence Lamarche, Hélène Ammann, Gabriel Dallaire, Louis Deslauriers, Stéphan Troyanov

**Affiliations:** Department of Medicine, Nephrology Service, Hôpital du Sacré-Coeur de Montréal, University of Montréal, Montréal, Quebec, Canada; Department of Medicine, Biochemistry Service, Hôpital du Sacré-Coeur de Montréal, University of Montréal, Montréal, Quebec, Canada; Department of Pharmacy, Hôpital du Sacré-Coeur de Montréal, University of Montréal, Montréal, Quebec, Canada; Department of Pharmacy, Hôpital du Sacré-Coeur de Montréal, University of Montréal, Montréal, Quebec, Canada; Department of Medicine, Nephrology Service, Hôpital du Sacré-Coeur de Montréal, University of Montréal, Montréal, Quebec, Canada

**Keywords:** desmopressin, hyponatremia, overcorrections, retrospective cohort study

## Abstract

**Background:**

The suggested narrow rate of serum sodium (sNa) correction in hyponatremia can be difficult to respect, leading to overcorrections. Our ability to anticipate the rapidity of correction according to the mechanism of hyponatremia is uncertain. While desmopressin is often used to pause a rapid rise in sNa, its dose-related effect is also not well described. We studied the rate of hyponatremia overcorrections, its prediction and the utility of desmopressin in its management.

**Methods:**

We retrospectively reviewed all cases of severe hyponatremia (sNa <120 mmol/L) in a large university hospital that occurred over 10 years. We assessed investigations, causes and treatments. We compared all sNa separated by at least 8 h and calculated correction rates. Significant overcorrection rates were defined by any rise of sNa >9 mmol/L per day sustained over at least 24 h.

**Results:**

After exclusions, we found 355 episodes of severe hyponatremia. Low, appropriate and inappropriate antidiuretic hormone (ADH)-defined mechanisms accounted for 17%, 24% and 29% of etiologies, respectively, with the remaining 25% secondary to diuretics and 5% of uncertain causes. First urinary sodium and osmolality were consistent with the final diagnosis in 73%. Significant overcorrections were seen in 45% and were frequent in the setting of low ADH. Desmopressin was given in 82 episodes, more often as a rescue than a preventive measure, with the subsequent sNa dropping by ≥5 mmol/L by 12 h in eight instances. The dose of desmopressin (≥2 µg versus 1 µg) and a higher volume of intravenous free-water coadministration resulted in a clinically meaningful greater reduction in sNa in the following 12 h.

**Conclusions:**

Overcorrections in severe hyponatremia are common, mainly when ADH is low. Initial urinary measurements anticipate this risk. Desmopressin effectively halted the rate of correction in a dose-dependent manner. Caution should be given when coadministrating water, which can significantly lower the sNa.

## Graphical Abstract

**Graphical Abstract:**
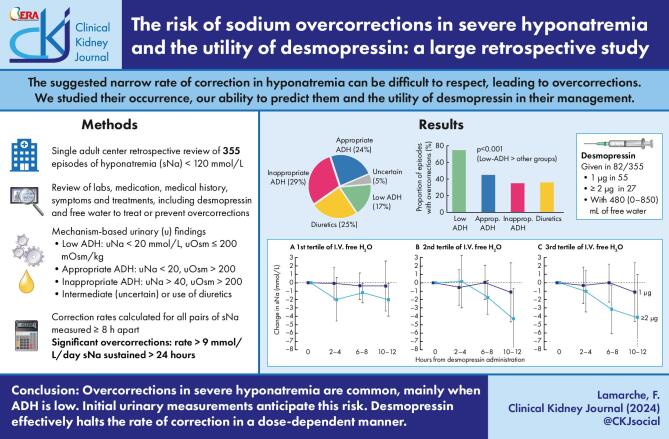

KEY LEARNING POINTS **What was known:**

- Overcorrections in hyponatremia carry the risk of osmotic demyelination syndrome.
- Desmopressin has been used to slow the rate of correction, but its dose-related effect is not well-described.

**This study adds:**

- Sustained overcorrections of hyponatremia are common, mainly when antidiuretic hormone is low and during recovery from acute kidney injury.
- First assessment of urinary sodium and osmolality allows us to anticipate overcorrections. Their early reassessment increases our predictive ability.
- Desmopressin is efficacious in avoiding a further rise in sodium. It is mainly used as a rescue therapy once overcorrections have occurred and can lead to clinically significant reductions in natremia with high water coadministration.

**Potential impact:**

- This study supports the preventive use of desmopressin when the risk of overcorrection is elevated.
- Caution should be given with the coadministration of water to treat overcorrections.
- These findings can simplify patient care, reduce overcorrections in hyponatremia and potentially improve patient outcomes.

## INTRODUCTION

Severe hyponatremia, defined by serum sodium (sNa) <120 mmol/L, poses a therapeutic challenge. A correction that is too slow is dangerous when neurological symptoms exist, whereas a rapid increase can lead to osmotic demyelination syndrome (ODS) [1]. Risk factors of ODS include chronic alcohol intake, hypokalemia, liver disease, malnutrition and initial sNa ≤105 mmol/L [2]. The American Expert Panel recommends a correction of sNa of 10–12 mmol/L over the first 24 h and of 18 mmol/L over 48 h for patients at average risk of ODS, and limiting the increase to 8 mmol/L over 24 h for patients at high risk of ODS [3]. However, ODS can occur despite adhering to these thresholds, mainly when risk factors are present [4]. Thus, a slower correction of 4–6 mmol/L per day for those with severe hyponatremia has been advocated [5].

This narrow window can be challenging to respect, given the various causes of hyponatremia, each associated with different spontaneous sNa evolutions. Low, appropriate and inappropriate antidiuretic hormone (ADH) mechanisms entail different treatment strategies. Urinary sodium (uNa) and osmolality (uOsm) help determine the etiology of hyponatremia, which in turn forecast the correction rate and influences treatments administered. However, urinary measurements can vary over time, and their initial predictive value is uncertain.

Upon rapid sNa correction, desmopressin (1-deamino-8-d-arginine vasopressin, DDAVP) reduces urinary water excretion and can slow or halt the rate of sNa correction. It is administered as proactive or rescue therapy and in a single or repeated dose [6–8]. Despite its use, the optimal administration of DDAVP for the prevention and treatment of overcorrection of hyponatremia has yet to be determined.

We retrospectively studied all severe episodes of hyponatremia that occurred over 10 years in a large university tertiary care hospital. We reviewed assessments, causes and treatments. Our first objective was to assess the causes of hyponatremia. We then determined the reliability of the initial urinary measurements to establish the final diagnosis. Third, we determined the risk of overcorrections according to the underlying mechanisms. Finally, we characterized how DDAVP administrations, with or without the coadministration of intravenous (IV) free water, halted sNa overcorrections. We hypothesize that overcorrections are frequent in low ADH states and that the initial urinary measurements predict the risk of overcorrections. We also postulate that DDAVP is an effective and straightforward method to treat or prevent overcorrections.

## MATERIALS AND METHODS

### Study design

We retrospectively reviewed all episodes of severe hyponatremia, defined by sNa <120 mmol/L, from January 2012 to June 2022. The ethics review board approved this study. We followed the Strengthening the Reporting of Observational Studies in Epidemiology (STROBE) guidelines. This work was carried out following the Helsinki Declaration.

### Population and data gathered

Using our central laboratory database, we extracted all measurements of sNa <120 mmol/L and all subsequent sNa in the following 10 days. Each studied episode required at least a second measurement ≥24 h. In each event, we recorded all available serum creatinine, urea, measured osmolality (sOsm), glucose, ethanol, cortisol and thyroid stimulating hormone (TSH) measurements, as well as any uNa and uOsm. We calculated osmolality using 2 * sNa + glucose + urea. We report first and second urinary measurements if done within 48 h. We excluded hyperosmolar states and obvious spurious measurements. Charts were reviewed (F.L. or S.T.) for a history of congestive heart failure, cirrhosis, malnutrition, alcohol intake or recent surgery. We recorded the occurrence of falls, confusion or convulsions at presentation and the presence and cause of hypovolemia. We reviewed medications at the time of diagnosis and during the hospitalization, including diuretics, desmopressin (initial dose, method of administration, repeated or single), and the administration of IV hypotonic, isotonic or hypertonic (NaCl 3%) fluids. We also documented oral administration of sodium and medicinal urea [9]. We could not assess oral water intake retrospectively. Finally, we report ODS and cause of death.

### Definitions

A uOsm >200 mOsm/kg denoted the presence of ADH [10]. This is a stricter definition than the classical Bartter and Schwartz criteria [11] (uOsm >100), given our older population where the maximal dilution capacity may be impaired [12]. Urinary sodium and osmolality at presentation allowed us to establish a presumed mechanism of hyponatremia. We categorized episodes into low ADH (uNa <20 mmol/L and uOsm ≤200), appropriate ADH (uNa <20 and uOsm >200), inappropriate ADH (uNa >40 and uOsm >200), intermediate criteria, and uninterpretable because of diuretics [13].

Appropriate ADH was further subdivided into true hypovolemia (e.g. diarrhea, adrenal insufficiency) and relative hypovolemia (i.e. reduced effective fluid volume in congestive heart failure and cirrhosis). “Tea and toast” and “beer potomania” required low ADH urinary measurements in a subject with a low osmolar diet or high alcohol intake, respectively [14, 15]. Syndrome of inappropriate ADH secretion (SIADH) required inappropriate ADH urinary assessment with normal thyroid and cortisol tests [16].

We established a final etiology of hyponatremia considering the history, medications upon arrival, daily clinical, further blood and urinary assessments, and response to therapy. Occasionally, the initial presumed mechanism differed from the final retained etiology [17]. If the final diagnosis from records appeared contradictory to the reviewer's interpretation, the second reviewer (F.L. or S.T.) analyzed the file and adjudicated the final diagnosis.

For each case of hyponatremia, we calculated the difference between every possible pair of sNa separated by a minimum of 8 h (Supplementary data, Fig. S1). The variations observed were reported as a rate over 24 h. Of all the different rates calculated, we report the most rapid correction speed (≤6, 7–9 and >9 mmol/L per day) and whether it was maintained for <1, 1–2 or ≥2 days. We defined a clinically significant overcorrection by a correction rate >9 mmol/L per day sustained over at least 24 h.

The volume of IV free water was calculated only for the 12 h following the first administration of desmopressin. For example, if a patient received during that time 500 mL of D5% H_2_O, D5% NaCl 0.45% or NaCl 0.9%, the equivalent free water given would be 500, 250 and 0 mL, respectively. Desmopressin administration following an overcorrection and with the coadministration of IV free water defined rescue therapy as opposed to a proactive or preventive treatment.

We report the presence of stage 1 and 2 acute kidney injury (AKI) using the KDIGO creatinine criteria [18]. Baseline creatinine was either the last measurement within 12 months before admission or the lowest creatinine observed during the hospitalization if corresponding to an estimated glomerular filtration rate (eGFR) >60 mL/min/1.73 m^2^, using the Chronic Kidney Disease Epidemiology Collaboration 2021 formula. A decrease in creatinine during the hospitalization to a level <150% of baseline defined recovery from AKI [19].

### Statistical analyses

Normally distributed variables are presented as means ± standard deviation. Nonparametric variables are expressed as medians with interquartile range. Categorical variables are summarized using proportions and are compared using the Pearson chi-square test.

We categorized the change in sNa (delta sNa) at 2–4, 6–8 and 10–12 h following the first desmopressin administration. We derived a linear mixed model to assess the influence of the dose of desmopressin and the volume of IV free water given in the first 12 h, expressed in tertiles, on the repeated sNa time points. For simplicity, we used a model with no intercept, given that the delta sNa at the time of administration was, by definition, 0. We studied fixed effects only, and given that the changes in sNa were likely to increase over time depending on the treatments administered, we used a first-order autoregressive structure with heterogeneous variances.

All *P*-values were two-tailed, and values <.05 were considered statistically significant. Confidence intervals included 95% of predicted values. SPSS 27 statistical software (IBM Corporation) was used for all analyses.

## RESULTS

### Patient selection

There were 419 episodes with sNa <120 mmol/L. Upon review, we excluded 18 events where the clinical file could not be found, 9 during hyperosmolar conditions, 31 cases defined by spurious outliers, 4 in subjects undergoing renal replacement therapy and 2 during mannitol therapy, leaving 355 episodes in 330 subjects. Fifteen patients had two distinct episodes, three had three, and one had five. Results are presented by episode but are unchanged if we consider only a single event per patient.

### Available and missing data

Each episode had a median of 19 (13–26) sNa measurements spread over 7 (5–8) days. There were 55 episodes where the sOsm was not measured, but in all of these, the calculated sOsm was reduced, even in 11/55 with ethanol intoxication and in 11/55 diabetic subjects. There were 10 cases where the serum osmolality was ≥280 mOsm/kg, 9 of which were accounted for by elevated urea and one by elevated ethanol. All episodes normalized their natremia with treatment, supporting absent hyperproteinemia or hyperlipidemia. Urinary measurements were available in 323/355 (91%) episodes at presentation and were repeated in 86/355 (24%) within the first 48 h. A TSH was measured in 270 episodes, and cortisol in 166. The baseline creatinine, which was necessary to determine the presence of AKI, could not be determined in three episodes.

### Cohort characteristics

At presentation, the age was 70 ± 15, 53% were female, and the initial sNa was 116 (113–118) mmol/L (Table 1). A past medical history of diabetes, alcohol abuse, heart failure and cirrhosis were present in 16%, 22%, 13% and 8.5%, respectively. Apparent malnutrition existed in 12%; 46% were on a diuretic and 3% had recent surgery. Baseline eGFR was 95 ± 24 mL/min/1.73 m^2^, with 28 (7.9%) <60 mL/min/1.73 m^2^. Sixty-three (18%) episodes presented stage 1 AKI, and 9.4% had stage 2. All but two AKI episodes recovered. Neurological symptoms of hyponatremia were frequent: 15% suffered a fall before their admission, 40% presented confusion and 6% had a seizure.

**Table 1:** Clinical characteristics at onset of severe hyponatremia episodes.

Variable	*N* = 355
Age (years)	70 ± 15
Female, *n* (%)	188 (53)
sNa (mmol/L)	116 (113–118)
Baseline eGFR (mL/min/1.73 m^2^)	95 ± 24
AKI, *n* (%)^a^	
Stage 1	63 (18)
Stage 2	33 (9.4)
Past medical history, *n* (%)	
Diabetes	67 (16)
Heart failure	45 (13)
Cirrhosis	30 (8.5)
Suspected malnutrition	41 (12)
Alcohol abuse	78 (22)
Recent surgery	12 (3.4)
Diuretics at presentation, *n* (%)	
Any	162 (46)
Loop	57 (16)
Thiazides	115 (32)
Distal	33 (9.3)
Neurological symptoms at presentation, *n* (%)	
Fall or ataxia	53 (15)
Confusion or delirium	144 (41)
Convulsion	22 (6.2)

Data are presented as mean ± standard deviation, median (interquartile range) or *n* (%).

For neurological data, if there was no mention in the file, we assumed the absence of manifestation.

a No data was missing except for baseline eGFR and AKI, which could not be determined in three episodes.

### Etiologies of hyponatremia

**Figure 1:**
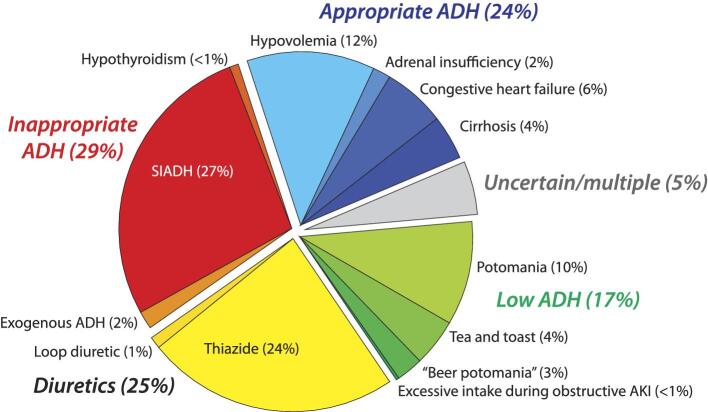
Causes of hyponatremia. Italic refers to the mechanism of hyponatremia.

The mechanisms and etiologies of hyponatremia are shown in Fig. 1. In the low ADH group (17%), the etiologies were 37 potomania (10%), 15 “tea and toast” syndrome (4%), 8 “beer potomania” (3%) and, in one case, high water intake with obstructive AKI. In the inappropriate ADH group (29%), we found 2 episodes with hypothyroidism (TSH 20.1 and 24.7 without another cause), 6 in the setting of exogenous DDAVP, and the remaining 97 (27%) were secondary to SIADH. Within this last group, 24/97 occurred secondary to drugs, 10 postoperatively or secondary to intense pain (± narcotics), 17 with pulmonary conditions (e.g. pneumonia), 14 with active cancer, 14 with neurological conditions and 18 without an apparent cause. In the appropriate ADH group (24%), we found 49 (14%) with true hypovolemia, including two cases of adrenal insufficiency. There were 35 events (10%) with relative hypovolemia, 14 with cirrhosis and 21 with congestive heart failure. As expected, AKI at presentation was more likely in the appropriate ADH group (Supplementary data, Fig. S2).

There were 18 episodes (5.0%) where no predominant causes could be established, and in 12 (3.3%), the most plausible final diagnostic differed between the chart and reviewers.

### Urinary measurements agreement with final retained diagnosis

Excluding urinary measurements that were unavailable, performed on diuretics or with intermediate findings, the initial urinary sodium and osmolality concorded with the final mechanism in 98/134 (73%) (Table 2). If a second urinary measurement within 48 h was concordant with the first assessment, this rose to 38/44 (86%). However, if the second measurements were inconsistent with the initial evaluation, the positive predictive value of the first measurements fell to 35% (6/17).

**Table 2:** Initial uNa and uOsm measurements concordance with final hyponatremia mechanism.

1st measurement, *n* (%)	2nd measurement^a^, *n*	Final retained mechanism, *n*	Positive predictive value of 1st urinary measurement, *n* (%)
Low ADH 32 (9)	Not measured: 16	Low ADH: 22	22/32 (69%)
uNa <20 mmol/L	Low ADH: 8^c^	Appropriate ADH: 5	- With concordant 2nd measurement: 7/8^c^ (88%)
uOsm ≤200 mOsm/kg	Appropriate ADH: 4^d^	Inappropriate ADH: 5	- With discordant 2nd measurement: 3/6 (50%)^d^
	Inappropriate ADH: 2^d^		
	Intermediate: 2		
Appropriate ADH 48 (14)	Not measured: 23	Low ADH: 5	32/48 (66%)
uNa <20 mmol/L	Low ADH: 2^d^	Appropriate ADH: 32	- With concordant 2nd measurement: 14/16^c^ (88%)
uOsm >200 mOsm/kg	Appropriate ADH:16^c^	Inappropriate ADH: 8	- With discordant 2nd measurement: 1/7 (14%)^d^
	Inappropriate ADH: 5^d^	Multiple mechanisms: 3	
	Diuretic given: 1		
	Intermediate: 1		
Inappropriate ADH 54 (15)	Not measured: 26	Low ADH: 4	1st measurement only: 44/54 (81%)
uNa >40 mmol/L	Low ADH: 2^d^	Appropriate ADH: 5	- With concordant 2nd measurements: 17/20^c^ (85%)
uOsm >200 mOsm/kg	Appropriate ADH: 2^d^	Inappropriate ADH: 44	- With discordant 2nd measurement: 2/4 (50%)^d^
	Inappropriate ADH: 20^c^	Multiple mechanism: 1	
	Diuretic given: 2		
	Intermediate: 2		
Diuretic 159 (45)			^b^
Intermediate findings 30 (8)	Not measured: 13	Low ADH: 12	^b^
	Low ADH: 5	Appropriate ADH: 5	
	Appropriate ADH: 2	Inappropriate ADH: 13	
	Inappropriate ADH: 4		
	Intermediate: 6		
Not measured 32 (9)			^b^

^a^The second measurement used the same urinary thresholds for categorization and must have been performed within 48 h.

^b^No positive predictive value could be established because the first urinary measurement did not fit the low, appropriate or inappropriate ADH groups.

^c^Concordant second urinary measurement.

^d^Discordant second urinary measurement (excluding those with intermediate findings on the second measurement).

### Treatment and Overcorrections

Table 3 shows the treatments offered. They varied extensively according to the underlying etiologies. NaCl 3% was given in 110 episodes, 19 of which seizures were observed or strongly suspected. In the remaining 91 episodes, 38 (42%) presented other neurological symptoms, and 53 (58%) had no neurological symptom.

**Table 3:** Treatments administered based on final diagnosis.

Final mechanistic etiology, *n* (% of all episodes)	Hypotonic IV fluids, *n* (%)	Isotonic IV fluids, *n* (%)	IV NaCl 3%, *n* (%)	Oral NaCl, *n* (%)	Oral urea, *n* (%)	Loop diuretic, *n* (%)	DDAVP^*^, *n* (%)
Low ADH^a^, 60 (17)	29 (48)	39 (65)	17 (28)	18 (30)	0 (0)	11 (18)	27 (45)
Appropriate ADH 84 (24)							
True hypovolemia^b^, 49 (14)	27 (55)	41 (84)	7 (14)	15 (31)	1 (2)	13 (29)	13 (27)
Effective hypovolemia^c^, 35 (10)	9 (26)	17 (49)	7 (20)	10 (29)	3 (9)	28 (80)	1 (3)
Inappropriate ADH^d^, 105 (29)	27 (26)	61 (58)	38 (36)	72 (69)	16 (15)	48 (46)	13 (12)
Diuretics^e^, 88 (25)	30 (34)	59 (67)	32 (36)	39 (44)	2 (2)	11 (13)	23 (26)
Uncertain or multiple, 18 (5)	6 (33)	11 (61)	9 (50)	9 (50)	1 (6)	7 (39)	5 (28)
*P*-value (excluding uncertain/multiple)	.01^(a,b>rest)^	.008^(b>rest)^	.03^(d,e>b)^	<.001^(d>rest)^	<.001^(d>rest)^	<.001^(c,d>rest)^	<.001^(a,b,e>c,d)^

No V2 receptor antagonists were used in this study.

^*^Excludes three patients receiving DDAVP for central diabetes insipidus. Comparisons between different groups were performed using Chi-square test, excluding hyponatremia of uncertain or multiple mechanisms.

**Figure 2:**
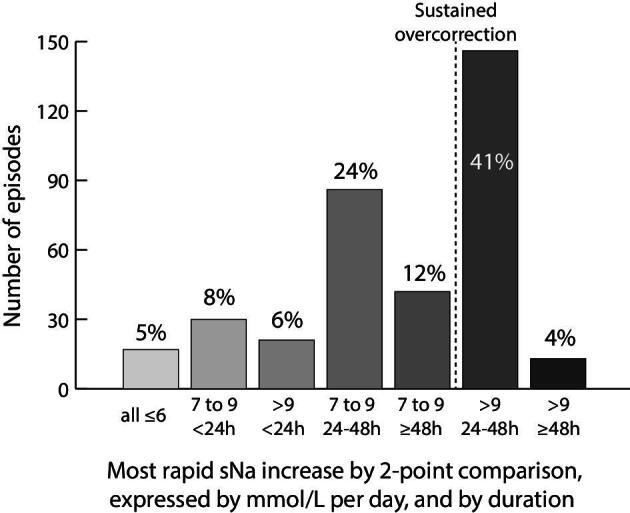
Most rapid rate of sodium correction per episode (*n* = 355). Significant overcorrections were defined by two-point measurements ≥24 h apart with a sNa correction rate >9 mmol/L per day.

**Figure 3:**
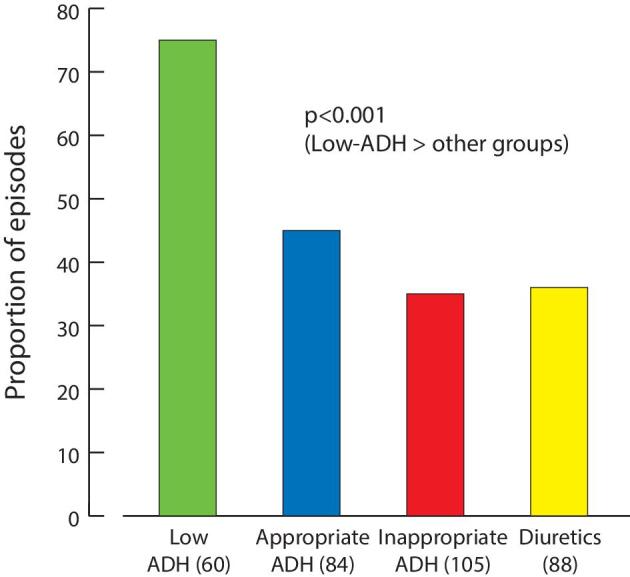
Sustained overcorrections according to the underlying mechanism of hyponatremia. Sustained overcorrections were defined by two-point measurements ≥24 h apart with a sNa correction rate > 9 mmol/L per day. The number of episodes in each category is in parentheses. Within the appropriate ADH groups, the proportion of overcorrections in true hypovolemia was similar to relative hypovolemia (46% vs 45%).

Only 5% of episodes had correction rates ≤6 mmol/L per day when considering every possible two-point sNa comparison (Fig. 2). Higher correction rates for <24 h accounted for 14% of cases, and increases of 7–9 mmol/L per day maintained over 24–48 h or ≥48 h accounted for 36% of cases. Finally, sustained overcorrections (>9 mmol/L per day maintained over 24–48 h or ≥48 h) existed in 45% of events and occurred early (Supplementary data, Fig. S3). They were observed in 75% of low ADH states, more often than other mechanisms (Fig. 3). The presence of AKI was marginally associated with a higher risk of overcorrection (56% vs 42%, *P *= .05). This was more evident in the true hypovolemia group (Supplementary data, Fig. S4).

### Treatment effects of desmopressin and coadministration of IV free water

Desmopressin was given in 82 episodes, excluding 3, where it was used to treat central diabetes insipidus. The first dose was 1 µg in 55/82 cases, 2 µg in 24, and 3 episodes received higher doses. In addition, it was administered subcutaneously in 66/82 cases, intravenously in 15 and intranasally in 1. Finally, it was repeated at least once in 31/82 instances. Desmopressin was often used as a rescue therapy once a significant overcorrection had occurred (Supplementary data, Fig. S5). The sNa had risen at a median rate of 11 (7–17) mmol/L per 24 h before its administration. It was given 19 (12–29) h after presentation, and individuals received 480 (0–850) mL of IV free water in the 12 h following the first dose. We found only six episodes with DDAVP given before any overcorrection.

**Figure 4:**
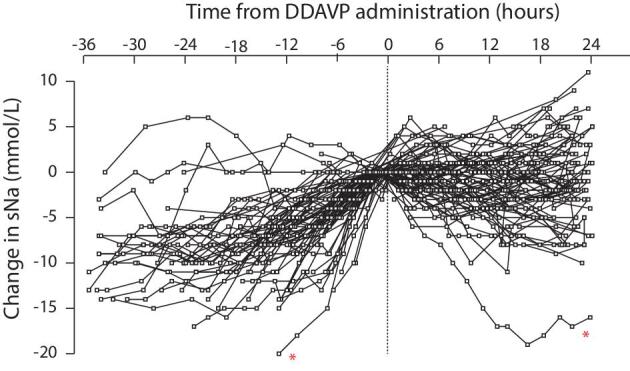
Evolution of serum sodium before and after DDAVP administration. ^*^In one exceptional case, there was a 20 mmol/L sNa rise over 12 h followed by a profound 19 mmol/L reduction over 18 h following multiple 2 µg desmopressin administration and >3 L of IV free water.

**Figure 5:**
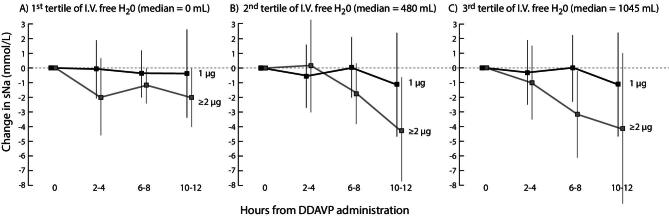
Change in serum sodium after different doses of DDAVP and administration of D5%. In 82 cases where desmopressin was administered, sNa were missing in 19, 12 and 11 episodes at the 2–4, 6–8 and 10–12 h time points. All other data was available.

Figure 4 illustrates the blunting of sNa rise following administration. The sNa dropped by ≥5 mmol/L in eight episodes. The 15 episodes where desmopressin was given intravenously experienced a similar change in sNa. Using a linear mixed model, higher desmopressin dose and free water volume resulted in a clinically meaningful greater reduction in sNa (Table 4). The additive effects of DDAVP and IV free-water administration are depicted in Fig. 5.

**Table 4:** Change in sNa following desmopressin administration.

Variables	Estimates of fixed effect on the change in sNa (mmol/L), with 95% CI	*P*-value
Desmopressin		
Dose 1 µg	–1.0 (–2.0, –0.0)	.04
Dose ≥2 µg	–2.8 (–3.7, –1.4)	<.001
Time of measurement following desmopressin administration		
2 to 4 h	+0.6 (0.0, 1.2)	.05
6 to 8 h	+0.3 (–0.2, 0.8)	.19
10 to 12 h	0^a^	
Tertiles of IV free water give in the first 12 h		
1st (median 0 mL)	+0.8 (–0.4, 2.0)	.19
2nd (median 480 mL)	+0.5 (-0.7, 1.6)	.44
3rd (median 1045 mL)	0^a^	

We used a linear mixed model with no intercept.

^a^These parameters were set to zero as a reference. Desmopressin, time and tertiles of IV free water were all statistically significant when studied univariately.

CI: confidence interval.

### Clinical outcomes

Seventeen (4.8%) patients died during hospitalization, none secondary to hyponatremia. Two cases of ODS confirmed by magnetic resonance imaging occurred. Both had alcoholic cirrhosis, hypokalemia and a sNa of 105 and 109 mmol/L at presentation, followed by maximal correction rates exceeding 9 mmol/L per day over 24–48 h. Both also received desmopressin, but only after the overcorrection had occurred.

## DISCUSSION

Rapid rises in sNa were common in this large retrospective cohort study, and sustained overcorrections reached 75% of low ADH states. Initial urinary measurements disagreed with the final diagnosis in 27%, and repeating their measurements within the first 48 h increased their predictive value. Desmopressin was almost always used as a rescue therapy rather than a preventive measure. It effectively slowed the correction rate in a dose-dependent manner, and the coadministration of IV free water further reduced sNa.

Our mechanistic definitions of hyponatremia deserve comment. Certain references support a >100 mOsm/kg urinary threshold to define the presence of ADH [3, 20]. However, our population was old, and the maximum dilution capacity may have been impaired [12]. Hence, we chose a higher threshold (>200 mOsm/kg) to increase the certainty of the presence of ADH. Nevertheless, even with a urine osmolality between 100 and 200 mOsm/kg, plasma ADH usually remains low (<1 ng/mL) [21]. We also used a >40 mmol/L uNa to define SIADH and maximize specificity, in contrast to references proposing a >20 or 30 mmol/L cut-off [20]. It is possible that we failed to identify some SIADH episodes, a difficulty often raised [22, 23], but our “uncertain or multiple mechanisms group” accounted for only 5% of all episodes.

Few studies have examined the predictive value of urinary findings in managing hyponatremia. As coexisting pain, nausea and vomiting can temporarily stimulate ADH secretion and mask a low ADH state, initial urinary measurements can be misleading. This highlights the importance of early reassessment of these parameters to reach an appropriate diagnosis and adjust treatments, as we showed that overcorrections occur very early [24]. While urinary electrolytes cannot be interpreted with diuretics, their use was associated with a greater desmopressin rescue strategy. Urinary measurements 24 h after they cease may unmask a low ADH state and facilitate management.

Desmopressin was used more often as a rescue therapy, as evidenced by the frequent coadministration of IV free water and the preceding overcorrection rate. Doubling the DDAVP dose approximately doubled the effect on sNa. Our findings are supported by a systematic review, where the amplitude of sNa lowering was –1.5 ± 0.7 mmol/L with a proactive strategy versus –8.2 ± 4.0 mmol/L with a rescue strategy [25]. This supports the idea that a proactive approach may be safer than a rescue strategy, avoiding significant sNa fluctuations [6, 7, 26]. However, desmopressin is not without risk, especially in individuals unwilling to respect water restrictions [27–29]. We found 8/82 episodes with a reduction of ≥5 mmol/L in sNa following its use, although this may have been intentional as clinical and experimental evidence support “un-correcting” overcorrections [30, 31]. While no seizures or other adverse events were found following the lowering of sNa, unwanted drops in sNa can prolong hospital stays and increase the number of interventions. Our 19 (13–26) sNa measurements spread over 7 (5–8) days highlight how resource-intensive the management of hyponatremia is.

Others also have identified frequent overcorrections [32]. Identifying patients at risk at their initial presentation can help guide management and reduce unnecessary treatments offered [23]. In this cohort, episodes with an “absent ADH” profile were at higher risk of overcorrection, in agreement with other studies [33]. Therefore, some overcorrections may have been iatrogenic, as exemplified by 28% of severe hyponatremias in low ADH states receiving NaCl 3% when many were asymptomatic. Interestingly, the prevalence of overcorrection in true hypovolemia was significantly higher when AKI coexisted. While a previous study reported no more rapid correction rates in those with AKI and hyponatremia, we postulate that these situations may benefit from a proactive use of DDAVP [34].

There has been debate on the appropriate rate of sodium correction; reports have been published where ODS occurred despite slow rises in sNa, leading to a slower correction rate being advocated [4]. In our study, ODS was rare and similar to recent cohorts [35]. Adding to this uncertainty, retrospective studies reported higher mortality in patients with a slower correction rate (<6 mmol/L per day) than in those with a faster rise and in those with higher sNa fluctuations [36, 37].

Our method of calculating the correction rate, which captured all measurements of sNa, is novel. This comes with the caveat that our calculation method may overestimate the incidence of overcorrections as some may have experienced an acute drop in sNa. However, all values were reviewed to eliminate spurious results. This study has limitations, starting with its retrospective nature, where confounding factors may have been missed. Multiple elements were at play, and the final etiology was inevitably subjective in some instances. For instance, spontaneous overcorrection might have led us to erroneously infer a low ADH state, mostly when urinary electrolyte data was missing, misleading us to conclude that “low ADH states increase the risk of overcorrections.” However, the final retained mechanism agreed with the initial urinary findings, and the high concordance between the initial and reviewers’ conclusions is reassuring. Another significant limitation of this study was our inability to assess water intake or urinary output.

In conclusion, overcorrections in severe hyponatremia are common, mainly when ADH is low. Urinary measurements anticipate this risk. Desmopressin effectively slows the rate of correction in a dose-dependent manner. Caution should be given when coadministering water, which can significantly lower the sNa. A proactive rather than rescue administration of desmopressin may prove safer and more straightforward.

## Supplementary Material

sfae386_Supplemental_File

## Data Availability

The data presented here will be shared upon reasonable request.
