# Supplementary material for: The risk of sodium overcorrections in severe hyponatremia and the utility of desmopressin: a large retrospective study
Source: Clin Kidney J. 2024 Nov 29;18(2):sfae386. doi: 10.1093/ckj/sfae386 (PMC11997796; doi:10.1093/ckj/sfae386)
Supplement: sfae386_Supplemental_File [file sfae386_Supplemental_File.docx]

**Supplemental Material**

**Supplemental Figure 1**: Example of correction rate calculation

**Supplemental Figure 2:** Acute kidney injury and mechanism of hyponatremia

**Supplemental Figure 3:** Time occurrence of sustained overcorrections

**Supplemental Figure 4**: Risk of a sustained overcorrection according to mechanisms of hyponatremia and presence of acute kidney injury.

**Supplemental Figure 5**: Desmopressin use according to the mechanism of hyponatremia and the presence of sustained overcorrection

**Supplemental Figure 1**: Example of correction rate calculations

**
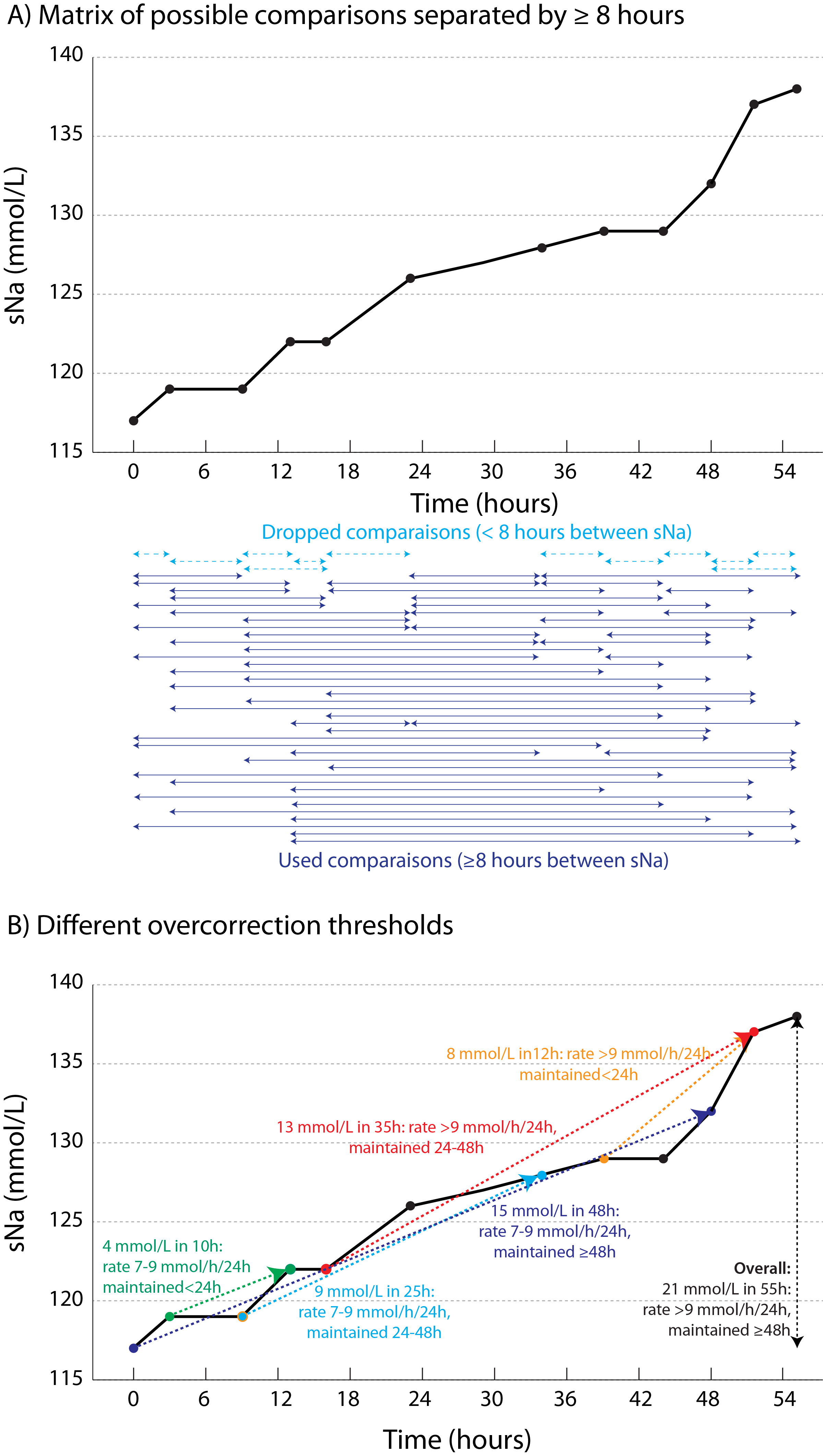
**

**Legend**: A) In pale blue, as opposed to dark blue, differences between any two measurements were too short (<8 hours) to estimate a 24-hour correction rate. These comparisons were discarded. B) Different overcorrections extrapolated over 24 hours, with duration. Sustained overcorrections were rates of> 9 mmol/L per day maintained 24 to 48 hours or ≥ 48 hours.

**Supplemental Figure 2:** Acute kidney injury and mechanism of hyponatremia


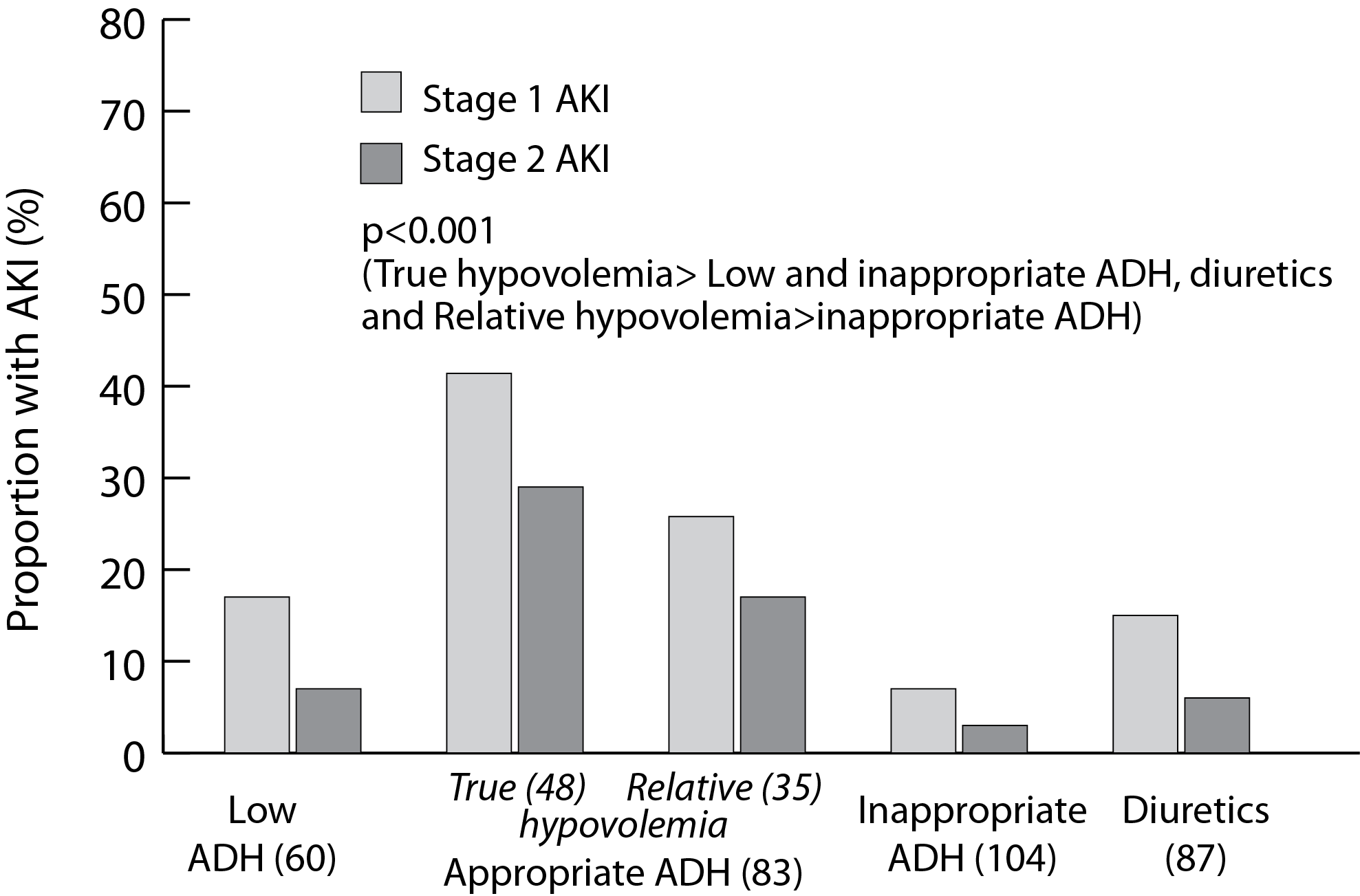


**Legend:** AKI, acute kidney injury defined according to KDIGO creatinine criteria. In 3 instances, we could not establish a baseline creatinine or the presence of AKI.

**Supplemental Figure 3:** Time occurrence of sustained overcorrections

**
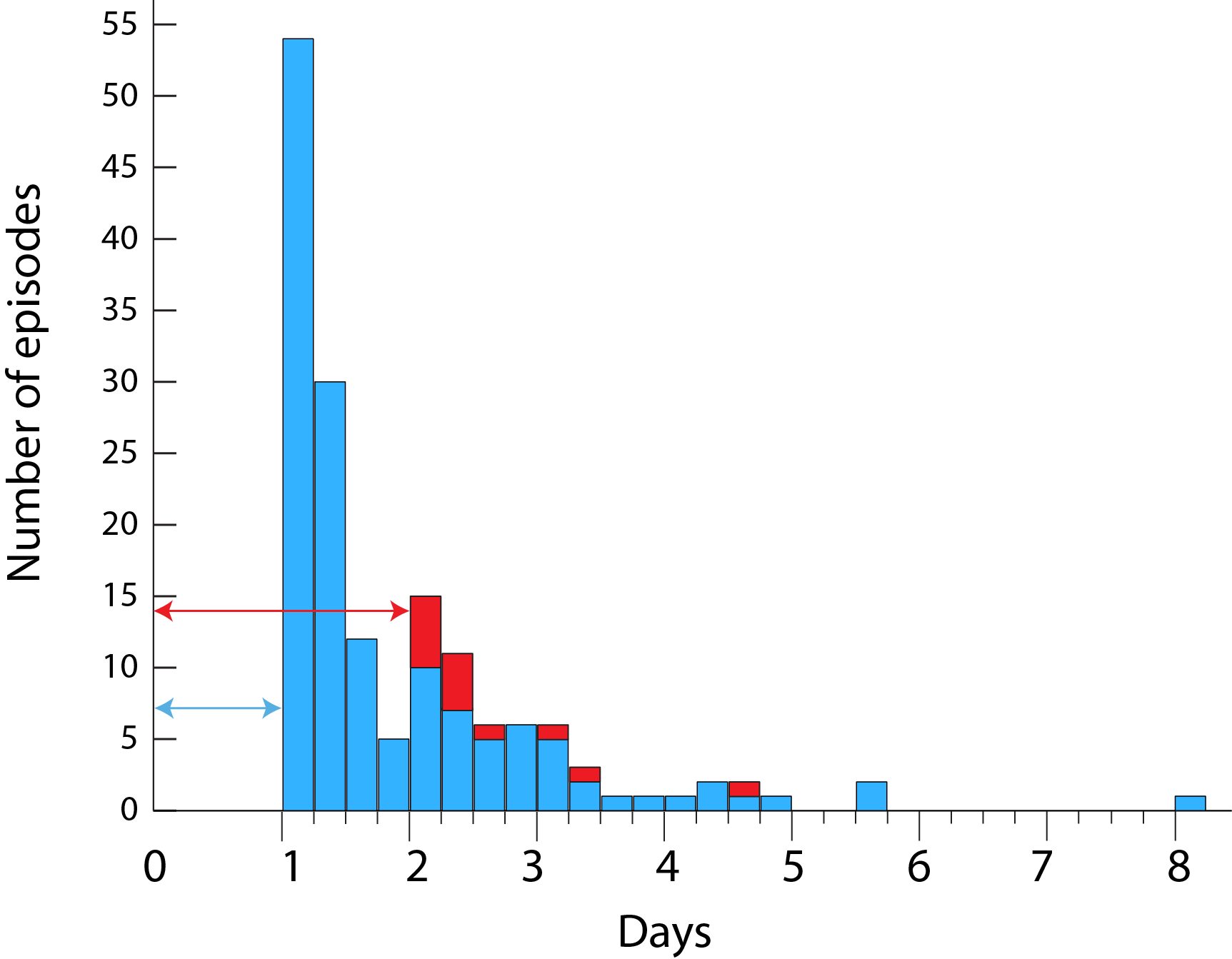
**

**Legend:** We defined a sustained overcorrection by a sNa increase >9 mmol/L per day sustained over at least 24 hours. Episodes where this rate was maintained for 1-2 and ≥ 2 days are in blue and red, respectively. The blue and red lines illustrate the minimal time required before identifying each overcorrection.

**Supplemental Figure 4**: Risk of a sustained overcorrection according to mechanisms of hyponatremia and presence of acute kidney injury.


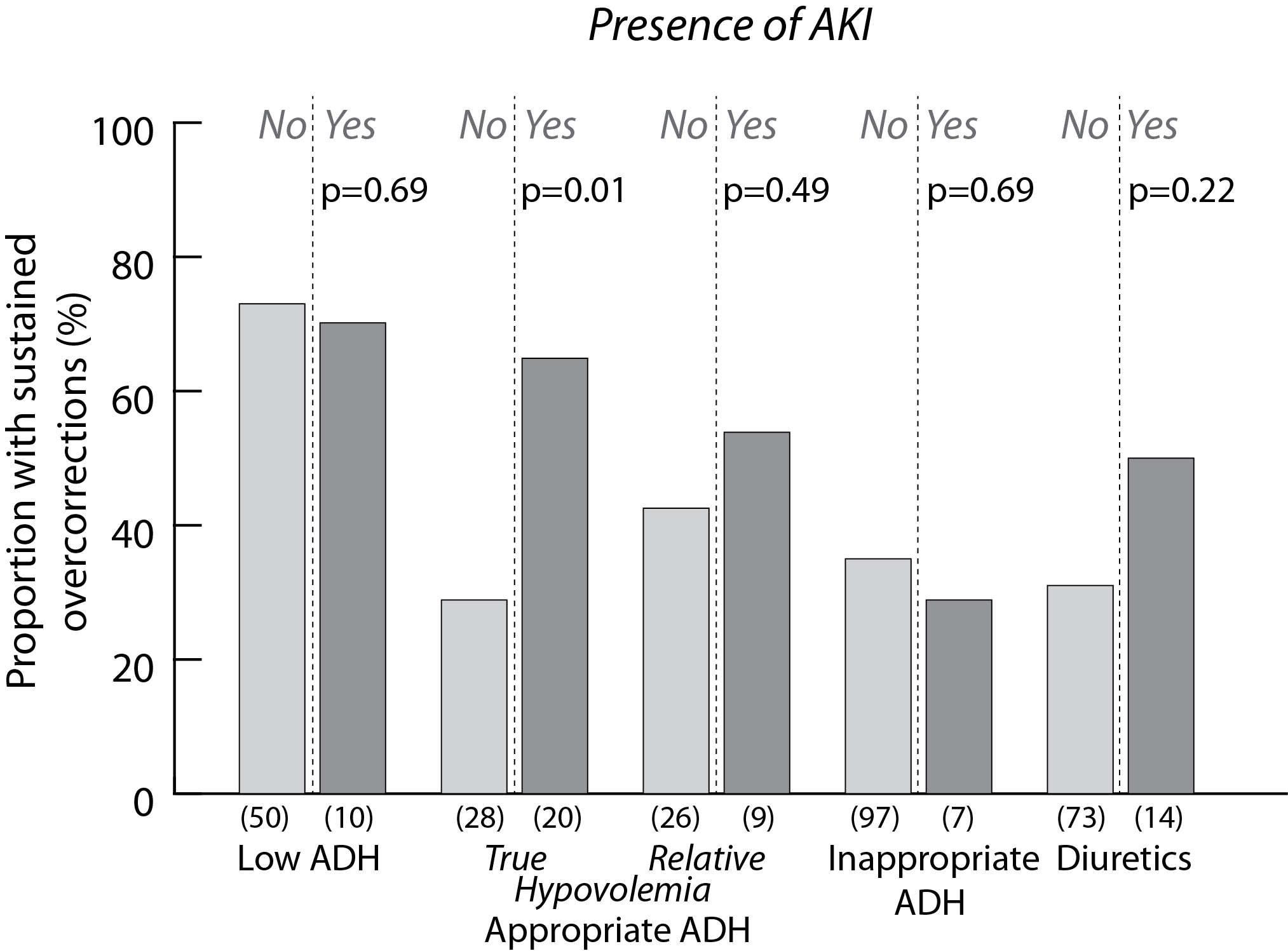


**Legend:** AKI, acute kidney injury defined according to stage 1 KDIGO creatinine criteria. In three instances, we could not establish a baseline creatinine or the presence of AKI.

**Supplemental Figure 5**: Desmopressin use according to the mechanism of hyponatremia and the presence of sustained overcorrection


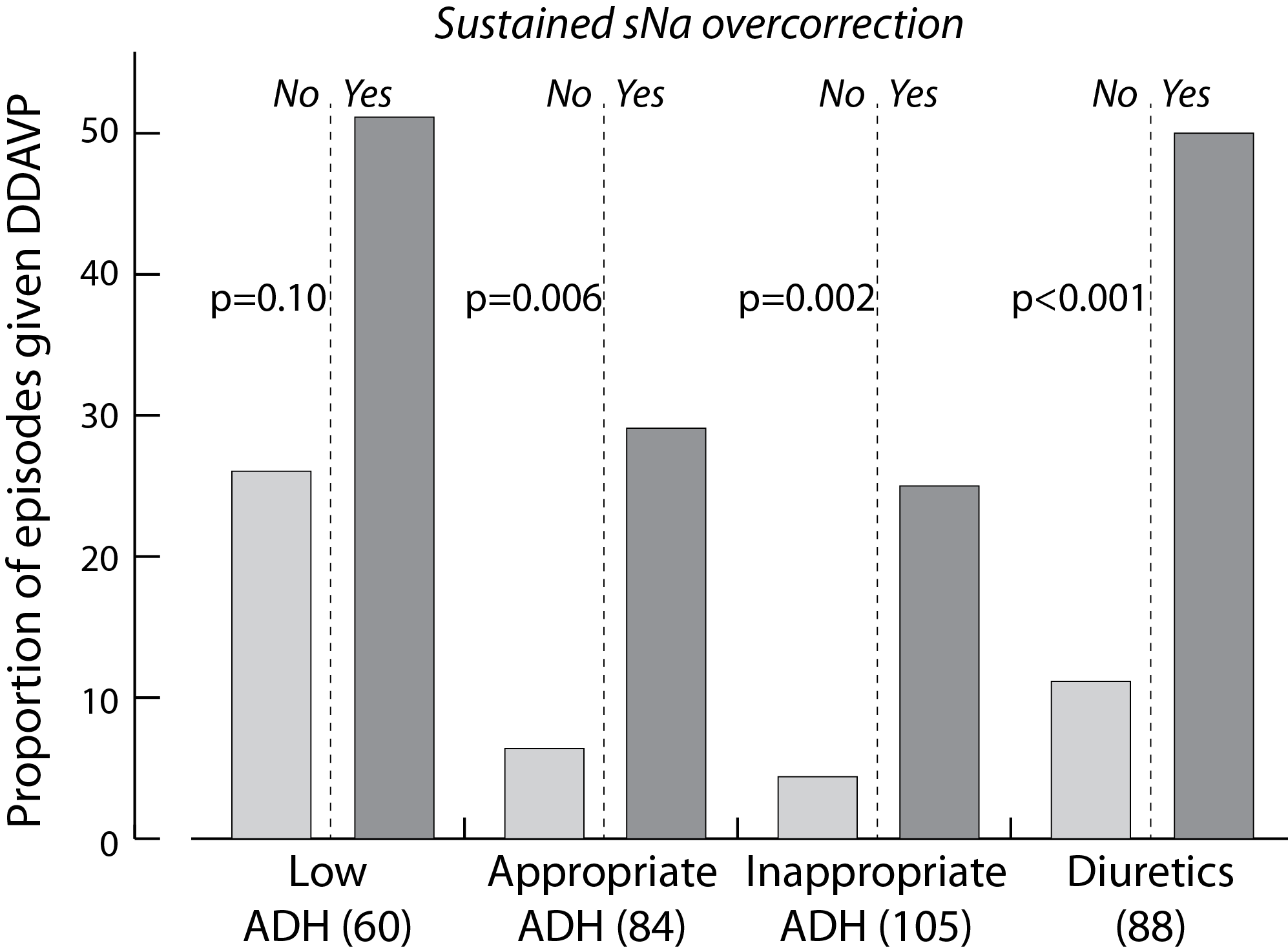


**Legend**: Desmopressin was administered more frequently in “low ADH” compared to each other categories (p<0.001) as well as in cases secondary to diuretics in comparison to “inappropriate ADH” (p=0.008). In addition, within each mechanistic category, DDAVP was given more often when overcorrection had occurred as rescue therapy rather than as a preventive measure.
